# Professional Change and Identity in Contemporary Physiotherapy: A Six‐Domain Conceptual Framework

**DOI:** 10.1002/pri.70367

**Published:** 2026-10-15

**Authors:** Dimitrios Stasinopoulos

**Affiliations:** ^1^ Department of Physiotherapy Faculty of Health and Caring Sciences Laboratory of Musculoskeletal Physiotherapy University of West Attica Athens Greece

**Keywords:** physiotherapy, professional change, professional development, professional identity

## Abstract

**Background and Purpose:**

Contemporary physiotherapy is often described as evidence‐informed, person‐centered, and biopsychosocial. These descriptions can imply a break with earlier practice, although some commitments have longer histories. This paper proposes a framework for examining professional change and its implications for professional identity.

**Methods:**

This single‐author conceptual analysis draws on a purposive reading of historical, sociological, critical, and professional literature. The professional maturation hypothesis is a working proposition: change may strengthen an enduring commitment or alter a foundational assumption. Contrasting examples inform six provisional domains; we did not conduct a systematic review or empirical validation.

**Results:**

The domains are understanding of the body and health, knowledge, professional role, therapeutic relationships, therapeutic purpose, and organization of care. Within each domain, the framework distinguishes maturation (better evidence or enactment), consolidation (institutional support), rebranding (new language without demonstrated substantive change), and rupture (a consequential shift in foundational assumptions). These interpretations may coexist.

**Discussion:**

The framework helps clinicians, educators, researchers, and professional organizations assess what has changed, where, to what extent, and with what consequences. Its intended use is context‐specific reflection and cross‐national inquiry, not classification of countries along a universal trajectory. The framework remains provisional and unvalidated.

## 1 Introduction

Physiotherapy is often presented as becoming more evidence‐informed, person‐centered, biopsychosocial, collaborative, and oriented toward active participation and self‐management. These commitments appear in professional descriptions of practice, education standards, and contemporary scholarship (World Confederation f and or Physical Therapy 2019; World Physiotherapy 2021; Nicholls et al. 2024). Professional identity scholarship examines the construction of legitimate work (Abbott 1988; Trede et al. 2012), while critical physiotherapy scholarship questions the reduction of earlier practice to bodily correction and technique (Nicholls and Gibson 2010; Nicholls 2017). The traditional–modern contrast considered here illustrates such professional narratives; however, its frequency in clinical or educational discourse has not been measured. Such contrasts may communicate dissatisfaction with ineffective, reductionist, or paternalistic care but can also oversimplify a diverse past.

Physiotherapy has changed substantially through research, evidence synthesis, professional autonomy, outcome measurement, digital delivery and interdisciplinary collaboration. The question is what these changes mean. Some developments strengthen earlier commitments; others change the assumptions on which care is based. The same development may have different implications across specialties and health systems.

Professional identity is partly shaped by accounts of a profession's past, present and future. These accounts influence professional authority, public recognition, education and the boundaries of acceptable practice (Abbott 1988; Freidson 2001; Trede et al. 2012). Describing change as a rupture can encourage reform but can also obscure continuity or overstate novelty. Equally, emphasizing continuity without scrutiny can minimize important changes or defend practices that need revision.

Critical physiotherapy scholarship has questioned familiar assumptions about bodies, normality, evidence, and professional identity (Nicholls and Gibson 2010; Nicholls 2012; Gibson 2016; Nicholls 2017; Nicholls et al. 2024). This paper proposes the professional maturation hypothesis as a starting point for comparison: some changes improve how an enduring purpose is understood or achieved, whereas others alter a foundational assumption. The framework also recognizes consolidation and rebranding. It examines six domains rather than issuing a single verdict about whether the profession is traditional or modern. Its practical aim is to make claims of change more specific and open to scrutiny (Figure 1).

**FIGURE 1:**
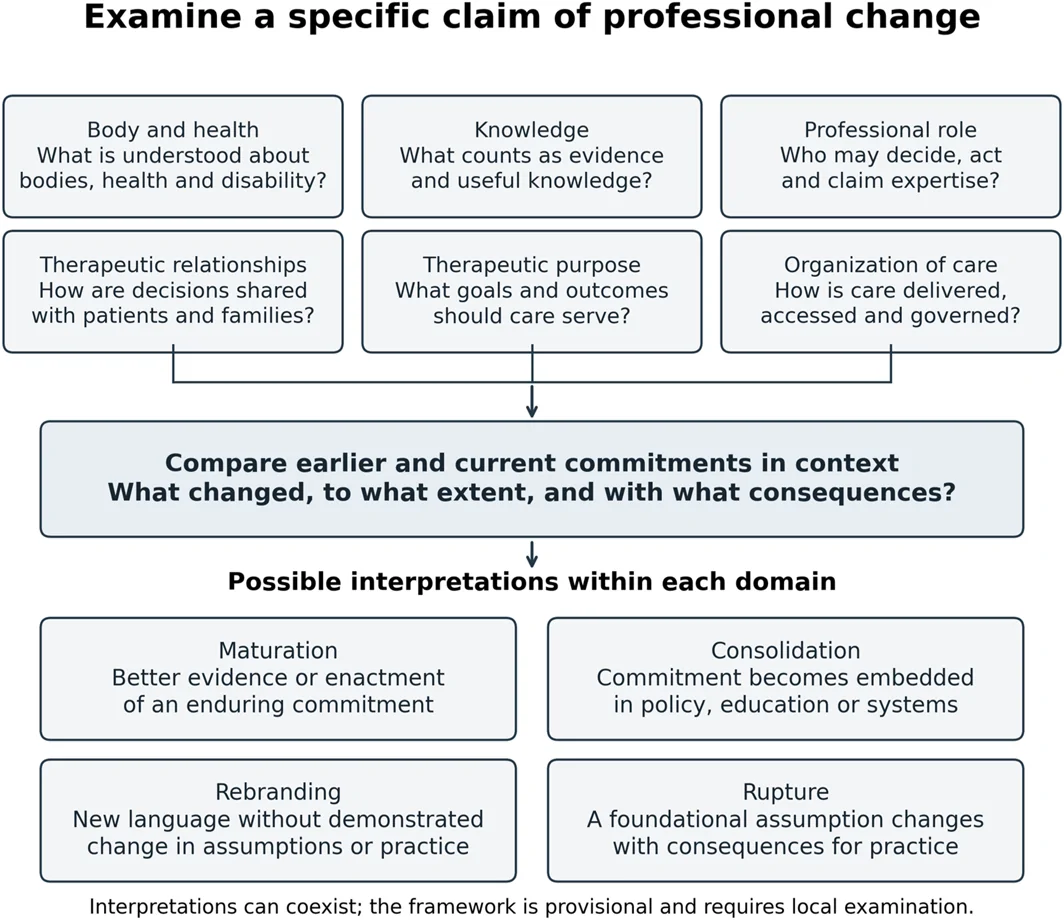
Six domains for examining professional change in physiotherapy. A specific claim is examined against earlier and current commitments in its context. Maturation, consolidation, rebranding, and rupture are possible interpretations within each domain and may coexist. The schematic provides a conceptual overview, not a validated decision rule or universal developmental sequence.

## 2 Methodological and Positionality Statement

This single‐author paper is a conceptual analysis. I compared historically grounded accounts of continuity with examples that challenge them. The approach accepts that interpretations may be mistaken and depend on context. Documents show what a profession claims to value; empirical research is needed to determine what happens in practice. Maturation and rupture are working distinctions informed by scholarship on paradigms, professions and critical physiotherapy (Kuhn 1962; Abbott 1988; Nicholls and Gibson 2010). They are not established theories of physiotherapy or categories derived from a coded dataset. I identified literature through prior reading, purposive searching, and citation tracking around professional history, identity, evidence‐based practice, direct access, and critiques of biomedical and biopsychosocial models.

I selected and interpreted the sources and developed the framework alone; there was no second coder or formal peer checking. Sources served four tasks: documenting professional commitments, explaining identity and authority, challenging assumptions, and illustrating contextual variation. Selection was purposeful and incomplete without a reproducible denominator for screening. I included critiques of research focused primarily on measurable effects and of biopsychosocial care reduced mainly to disease or bodily deficits. The six domains separate understandings of bodies and health (ontology), what counts as knowledge (epistemology), professional authority and scope (jurisdiction), how decisions are shared with patients, therapy goals, and service organization. These domains offer broad coverage but overlap in practice and remain open to revision (Figure 1).

This selective approach cannot estimate how common particular views are or provide a complete professional history. Modern physiotherapy has no single agreed meaning and often appears in informal communication that is difficult to study systematically. Here, the term is examined as a claim about professional identity and change rather than as a standardized model of care.

I am a physiotherapist, educator, and researcher working within a European university and healthcare context. This position offers experience of professional change but privileges European and musculoskeletal perspectives. I do not assume that the same sequence applies across specialties, countries, or Indigenous and non‐Western traditions. Examine the proposal locally and not as a universal chronology.

## 3 Defining Modernity, Rupture, Maturation and Rebranding

Modern physiotherapy has no universally agreed definition. Here, the term refers to claims that practice should be evidence‐informed, support active participation, consider the whole person, share decisions, measure outcomes and involve other professions. Traditional physiotherapy is a retrospective label for practices portrayed as passive, practitioner‐led, or focused mainly on biomedical explanations and treatment techniques. Neither label accurately describes all practice in a period or place. Both can obscure differences within the profession.

A paradigm is a set of assumptions about what a profession should study, how it should know, and what it should achieve (Kuhn 1962). Rupture means a consequential change in a foundational assumption, rather than simply a new technique or a broken profession. Change can be gradual and coexist with continuity in other domains. Figure 1 presents the six domains and shows how the four interpretations relate to them; Table 1 supplies the working criteria.

**TABLE 1:** Working distinctions for interpreting professional change.

Category	Working criterion	Illustrative indicator
Maturation	Better evidence or enactment of an enduring purpose	Outcome measurement for an established goal
Consolidation	Institutional stabilization of a commitment	Curriculum, guideline, or policy adoption
Rebranding	New language without demonstrated conceptual or practical change	Novel label for otherwise unchanged care
Rupture	Consequential shift in a foundational assumption	Changed authority, purpose or object of care

Maturation means better understanding, evidence, or implementation of an existing commitment. Consolidation embeds that commitment in curricula, guidelines, measures, or service structures. Rebranding means new terminology or positioning without demonstrated substantive change in assumptions or practice. These interpretations are not mutually exclusive: a service may consolidate a familiar purpose while changing who makes decisions or how people access care.

## 4 Professional Identity and the Construction of Modernity

Professions negotiate jurisdiction and legitimacy by narrating their expertise and social value (Abbott 1988; Freidson 2001). Modernization language can operate as description, aspiration, and boundary work: drawing or defending distinctions about who may claim expertise, perform tasks, or define legitimate practice. This interpretive reading of professional narratives is a proposed account and not a measured frequency of such claims.

Simple contrasts can make professional differences easy to describe while hiding variations in practice. Exercise can be prescribed without involving the patient; hands‐on care can support shared goals; and a treatment described as evidence‐based can overlook patient values. Therefore, a new label does not establish that care has changed.

Critical physiotherapy scholarship questions normalization—treating conformity to expected bodily standards as a therapeutic goal—as well as bodily correction, individualism, and the priority given to measurable knowledge (Nicholls and Gibson 2010; Nicholls 2012; Gibson 2016; Nicholls 2017). Reflection by the Critical Physiotherapy Network documents an international discussion of what physiotherapy is and might become (Nicholls et al. 2024). This framework provides criteria for examining how deeply proposed changes affect care.

## 5 Historical Continuities and Their Limits

Several commitments presented as contemporary were visible in earlier physiotherapy education and practice. By the mid‐twentieth century, therapeutic exercise was sufficiently established to be presented as a systematic component of rehabilitation rather than a recent alternative to passive treatment (Gardiner 1953). Clinical reasoning, functional assessment, goal‐directed planning, communication, and patient education also did not originate in the current century. Work on expertise in physiotherapy documented multidimensional knowledge, collaborative clinical reasoning, and attention to patients' lives rather than merely technical intervention (Jensen et al. 2000). Subsequent empirical work described the diagnostic, narrative, procedural, interactive, collaborative, and ethical reasoning strategies used by expert physiotherapists, further showing that clinical reasoning was not reducible to selecting a technique (Edwards et al. 2004). Therefore, the clinical reasoning literature formalized and made more explicit practices that had evolved within health‐professional education (Higgs and Jones 2000).

Historical continuity should not be romanticized: acknowledging earlier examples of exercise and collaborative reasoning does not imply that past practice was equitable or consistently patient‐centered—biomedical institutions, professional hierarchies, and normative understandings of bodies shaped earlier care. Physiotherapy's association with restoration and normal movement could marginalize people whose lives did not conform to expected bodily standards (Nicholls and Gibson 2010). Autonomy and participation varied by jurisdiction. The historical record contains multiple strands that disappear when the past is reduced to passive modalities.

The distinction between a longstanding commitment and its consistent enactment matters. A principle already articulated but later better theorized, measured, and disseminated may show substantial maturation. A changed therapeutic purpose, such as shifting from bodily normalization toward support for plural ways of living, may instead indicate a deeper conceptual departure.

## 6 Evidence‐Based Practice Maturation and the Limits of Positivism

Evidence‐based practice is defined as integrating the best available research evidence with clinical expertise and patient values (Sackett et al. 1996). Incorporating it into physiotherapy education and developing discipline‐specific resources greatly strengthened access to trials, reviews, and guidelines (Herbert et al. 2011; Iles and Davidson 2006). Implementation research, stratified models of care, and routine outcome measurement further created an infrastructure through which evidence could influence decisions (Foster et al. 2013; Greenhalgh and Wieringa 2011; Scurlock‐Evans et al. 2014).

These developments strongly reflect maturation and consolidation: they make clinical claims more testable, reduce reliance on authority, and create mechanisms for accountability. They do not, by themselves, replace the profession's longstanding purposes of improving function, reducing disability and enabling participation.

Evidence‐based practice may nevertheless be interpreted too narrowly, with experimentally measured effects taking priority over lived experience and social context. Positivism, used here to describe an emphasis on observable measurement as the principal basis for knowledge, has been criticized for treating other ways of understanding care as secondary (Nicholls 2012; Gibson 2016). Relational approaches instead emphasize connections among people, environments and institutions, rather than viewing a patient as an isolated body receiving treatment (Nicholls et al. 2016).

These critiques challenge a simple maturation account. Integrating research, expertise, values and context may strengthen the established purpose of evidence‐based practice. Changing how the profession understands bodies and legitimate knowledge may go further. The framework allows both possibilities to be examined in the body‐and‐health and knowledge domains.

## 7 The Biopsychosocial Model Between Expansion and Biomedicalisation

Engel's biopsychosocial proposal challenged biomedical reductionism by arguing that biological, psychological and social levels are dynamically implicated in health and illness (Engel 1977). Contemporary physiotherapy has increasingly incorporated psychological factors, pain education, self‐management and communication. This development has expanded the language and tools available for multidimensional care.

Adding psychological or social factors to an assessment does not necessarily change the model of care. In low back pain, the biopsychosocial model may be reduced to risk‐factor lists or attempts to correct patients' beliefs while wider social conditions receive less attention (Mescouto et al. 2022). Enactive approaches understand health through a person's ongoing interaction with the environment (Cormack et al. 2023). Ethical multiplicity means allowing more than one way to understand and respond to a person's needs, rather than expecting one model to explain every case (Mescouto et al. 2023).

The interpretation therefore depends on how care is delivered. New biopsychosocial language without changed assumptions or conduct may be rebranding. A wider assessment may extend existing practice. A changed understanding of the person and environment may involve a deeper departure. These are possibilities for examination and not a classification of all contemporary physiotherapy.

Health promotion and multimorbidity—coexisting health conditions—also challenge a history centered on musculoskeletal care. The global health Summits called for physiotherapy to address non‐communicable diseases across presenting complaints. The International Classification of Functioning, Disability and Health (ICF) considers functioning alongside personal and environmental contexts (Dean et al. 2011, 2014, 2019; World Health Organization 2001). These commitments predate some current modernization claims. How far they are incorporated into practice, and whether they extend earlier goals or change therapeutic purpose must be examined in each setting.

## 8 Examining the Professional Maturation Hypothesis

The proposed hypothesis expects maturation where developments improve the reliability, reach, or articulation of commitments already present in the profession. Examples include access to research, synthesis, outcome measures, shared goal setting, guidelines, and implementation strategies. These tools can improve the information available to clinicians and the consistency of care delivery while retaining longstanding purposes involving function, movement, participation, and education.

Counterexamples also matter. Direct access can change jurisdiction by positioning physiotherapists as first‐contact practitioners; disability scholarship can challenge normalization as a therapeutic end. Relational and enactive perspectives can question an ontology centered on the isolated body, while remote care can alter where and how therapeutic interaction occurs. These examples probe the hypothesis's explanatory adequacy; they are conceptual comparisons, not empirical tests of whether such shifts have occurred uniformly in routine practice.

The comparison does not support an absolute denial of rupture. Rather, the scope of each claim must be specified. Contemporary physiotherapy may combine continuity, maturation, rebranding and domain‐specific disruption. Each claim of novelty should identify its domain, depth, context and practical consequence.

Application starts with a specific claim of change. Which domain is affected? What do earlier and current documents, education, regulation, or routine care show? Does the change improve an existing commitment, embed it in a system, rename it, or alter an assumption with practical consequences? Who experiences those consequences? Figure 1 organizes these questions; Table 1 helps distinguish possible interpretations. Repeat the same assessment in each relevant context rather than generalizing across countries.

## 9 Illustrative Application of the Framework

Consider a hypothetical service introducing shared goal setting for people with persistent shoulder pain. A policy that formalizes patient participation while retaining the familiar goal of improved function may consolidate an existing commitment. If patients can also redefine the goals around participation despite persistent symptoms, the therapeutic purpose may change. Interviews, documents, and observed consultations would be needed to distinguish these possibilities. The distinction matters because a new policy alone does not change patients' influence or experience.

Two further hypothetical examples show how the same questions could be used beyond musculoskeletal care. In neurological rehabilitation, embedding patient‐selected participation goals in service reviews may reinforce existing person‐centered commitments; shifting away from normal movement as the overriding aim would require examining therapeutic purpose. In cardiorespiratory physiotherapy, adding remote follow‐up may change care organization while preserving established rehabilitation goals; access, family involvement, and responsibility for monitoring would need local assessment. Neither example establishes that a particular change has occurred across countries or specialties.

## 10 International and Contextual Boundaries

Professional change occurs within different regulatory systems, educational structures, service capacities and population needs. A global survey found variation in direct access and patient self‐referral (Bury and Stokes 2013). Scope, regulation, and entry‐level education also differ across jurisdictions (World Confederation for Physical Therapy 2019; World Physiotherapy 2021). Rehabilitation 2030 highlights unmet needs and under‐resourced services, especially in low‐ and middle‐income countries (World Health Organization 2017). A development that expands autonomy in one setting may already be established or remain unavailable in another. The present analysis cannot establish a universal sequence of professional development.

Modernization language can create hierarchy if well‐resourced institutions, particularly in the Global North, are treated as the endpoint for all professional development. Family participation, community services and local knowledge may matter differently across settings. Access to research, workforce shortages and referral requirements may also affect what is feasible. Cross‐national comparison should examine these differences without ranking countries by closeness to one preferred model.

The framework supports context‐specific and cross‐national examination. Researchers should ask whose history is being described, whose perspectives are absent, and whether an innovation is new to the profession, a jurisdiction, a specialty, or only a particular discussion. The European and musculoskeletal examples in this paper illustrate questions; they do not represent physiotherapy globally.

## 11 Future Research and Framework Development

Future studies could use historically diverse documents and patient and clinician accounts across specialties and countries with explicit source selection. Independent analysts could compare interpretations using Table 1, record disagreements and revise the domains or criteria. Tracking changes over time could assess whether claims of change match regulation, teaching, and consultations. These studies would evaluate usefulness, consistency and contextual limits without presuming that the present framework is valid.

## 12 Limitations

This selective analysis cannot establish how often professionals use labels or how care is delivered. English‐language sources and my European and musculoskeletal position underrepresent other histories and specialties. Global health scholarship and the ICF provide counterexamples but have not been comprehensively analyzed (Dean et al. 2011, 2014, 2019; World Health Organization 2001). The clinical examples are hypothetical and do not document adoption or outcomes in any country. The framework has not been validated; its categories may overlap, and language changes can precede changes in practice.

## 13 Conclusion

This paper contributes a structured way to examine professional change: what has changed, in which domain, to what extent, in what context, and with what consequences. Maturation, consolidation, rebranding and rupture can coexist within one development. Their interpretation requires evidence about earlier commitments and current practice rather than reliance on a modern or traditional label.

The six‐domain framework can make professional debate more precise and guide local reflection and cross‐national research. It does not establish a universal trajectory or rank specialties, generations or countries. Its proposed distinctions remain provisional; empirical and historical inquiry may support, revise, or reject them.

## 14 Implications for Physiotherapy Practice

For educators and students, the framework turns claims of novelty into questions that can be examined. A curriculum comparison can ask which domains have changed and whether a principle is new or has become better taught and supported. This helps preserve useful knowledge while identifying commitments that need reconsideration. The ICF and global health competencies support attention to participation, multimorbidity and context in such comparisons (World Health Organization 2001; Dean et al. 2019).

For clinicians, the framework helps distinguish a new label or policy from a change that patients experience. A shoulder‐pain service can examine whether adding comorbidity assessment strengthens existing care, whether shared goals are routinely used, and whether patients have greater influence over decisions. This can identify the need for implementation work, revised goals, or different decision‐making arrangements. The framework itself does not establish a treatment's effectiveness.

For researchers, distinguishing the types of change clarifies what evidence is needed. A claim of consolidation calls for evidence of institutional support; a claim of rupture requires examination of changed assumptions and their practical consequences. Document comparisons, interviews, observations, and cross‐national studies can compare professional claims with patient experience and routine care. Sampling beyond musculoskeletal and Global North settings is essential.

For professional organizations, the framework can clarify reform priorities. A familiar commitment may need resources and training, while a changed professional role may require revised governance. Machine‐learning tools illustrate this distinction: improved prediction may extend existing assessment, whereas changes in algorithmic authority or data governance may affect knowledge and service organization. Evidence gaps in validation and availability warrant caution (Reis et al. 2024). This is a potential application of the framework, not a claim that a global transformation has occurred.

## Funding

The author has nothing to report.

## Ethics Statement

The author has nothing to report.

## Consent

The author has nothing to report.

## Conflicts of Interest

The author declares no conflicts of interest.

## Data Availability

No new data were generated or analyzed for this conceptual article.
